# TGF-β based risk model to predict the prognosis and immune features in glioblastoma

**DOI:** 10.3389/fneur.2023.1188383

**Published:** 2023-06-29

**Authors:** Hongchao Liu, Zhihao Wei, Yu Zhang, Kangke Shi, Jiaqiong Li

**Affiliations:** Department of Pathology, The Yiluo Hospital of Luoyang, The Teaching Hospital of Henan University of Science and Technology, Luoyang, China

**Keywords:** glioblastoma, TGF-β, risk score, prognosis, immunotherapy, tumor microenvironment

## Abstract

**Background:**

Transforming growth factor-β (TGF-β) is a multifunctional cytokine with an important role in tissue development and tumorigenesis. TGF-β can inhibit the function of many immune cells, prevent T cells from penetrating into the tumor center, so that the tumor cells escape from immune surveillance and lead to low sensitivity to immunotherapy. However, its potential roles in predicting clinical prognosis and tumor microenvironment (TME) immune features need to be deeply investigated in glioblastoma (GBM).

**Methods:**

The TCGA-GBM dataset was obtained from the Cancer Genome Atlas, and the validation dataset was downloaded from Gene Expression Omnibus. Firstly, differentially expressed TGF-β genes (DEGs) were screened between GBM and normal samples. Then, univariate and multivariate Cox analyses were used to identify prognostic genes and develop the TGF-β risk model. Subsequently, the roles of TGF-β risk score in predicting clinical prognosis and immune characteristics were investigated.

**Results:**

The TGF-β risk score signature with an independent prognostic value was successfully developed. The TGF-β risk score was positively correlated with the infiltration levels of tumor-infiltrating immune cells, and the activities of anticancer immunity steps. In addition, the TGF-β risk score was positively related to the expression of immune checkpoints. Besides, the high score indicated higher sensitivity to immune checkpoint inhibitors.

**Conclusions:**

We first developed and validated a TGF-β risk signature that could predict the clinical prognosis and TME immune features for GBM. In addition, the TGF-β signature could guide a more personalized therapeutic approach for GBM.

## Introduction

Glioblastoma (GBM) is the most common malignant brain tumor. GBM patients generally have a poor prognosis, with a mean survival of approximately one year (1). At present, the main treatment used for GBM includes surgery, chemotherapy, radiotherapy, targeted therapy and immunotherapy (2, 3). Despite improvements in clinical treatment modalities, the prognosis for GBM remains dismal and GBM relapsed in almost all patients (4, 5). Therefore, potential biomarkers that can predict treatment response are needed urgently to promote personalized therapies for GBM.

Transforming growth factor-β (TGF-β) is a multifunctional cytokine involved in tissue development and tumorigenesis. TGF-β displays dual roles in both tumor suppression and tumor promotion during the tumor formation (6–8). TGF-β related genes were highly expressed in GBM and associated with poor outcome in this disease (9). TGF-β pathway influences various key processes in GBM progression such as stemness, migration/invasion, angiogenesis, immunosuppression, and drug/radio resistance (10). In addition, TGF-β pathway promotes immune escape of tumor cells and results in resistance to immunotherapy (11, 12). In consideration of the critical functions of TGF-β in regulating various tumorigenic processes, TGF-β antagonistic strategies are among the most promising of the innovative treatment approaches for GBM (13). A number of agents targeting TGF-β signaling are currently being investigated in several clinical trials of GBM patients (14, 15). At present, there is still no systematic research illustrating the TGF-β signature with the tumor microenvironment (TME) immune features in GBM.

In present work, we first developed and validated a novel TGF-β risk score model with several GBM datasets. The TGF-β risk score was strongly associated with clinical outcomes and TME immune features, and could evaluate the clinical response to chemotherapy for GBM.

## Materials and methods

### Datasets retrieval and data preprocessing

#### Training cohort

We first downloaded TCGA-GBM gene mutation data, RNA expression data, and clinical information from the Cancer Genome Atlas (TCGA) by using the ‘TCGAbiolinks’ R package (16). For GBM samples in the TCGA dataset, samples without survival status or survival time were excluded. After data preprocessing, the TCGA-GBM cohort included 143 primary GBM samples and five normal samples for further analysis. The clinical characteristics of patients were listed in Supplementary Table S1. Supplementary Figure S1 showed the workflow of this study.

#### Validation cohort

GSE121720 (58 GBM samples and four normal samples) dataset with expression profiles and clinical data, was obtained from the Gene Expression Omnibus (GEO) (17). The gene probes in GSE121720 dataset were transformed to gene symbols. Probes annotated to multiple genes were discarded, and the average value of multiple probes mapped to one gene was selected.

#### TGF-β related genes

TGF-β related genes were retrieved from the MSigDB (v7.4) with the following search terms: HALLMARK_TGF_BETA_SIGNALING and KEGG_TGF_BETA_SIGNALING_PATHWAY (18). In total, 121 TGF-β related genes were summarized after screening the original data (Supplementary Table S2).

#### Screening of differential TGF-β genes

We screened differentially expressed TGF-β genes (DEGs) between GBM and normal samples with the ‘limma’ R package (19). |log_2_ (fold change) | > 0.5 and corrected *p*-value less than 0.05 were set as the significance threshold. Then, the protein–protein interaction (PPI) network was constructed with the STRING database (20).

#### Construction and validation of the TGF-β risk model

Through univariate Cox analysis, DEGs that were related to GBM survival were screened in the TCGA-GBM dataset. Then, we performed multivariable Cox regression on the prognostic TGF-β DEGs. Then the TGF-β risk score model was developed based on the expression level and corresponding coefficient of each gene. The risk score of each sample was calculated according to the formula as follow: Risk score = Σ exp_i_*β_i_, where exp_i_ is the expression and β_i_ is the regression coefficient.

According to the formula, the risk score for each patient was calculated, and the patients were then classified into high-or low-score groups using the median risk score. Kaplan–Meier survival curves and log-rank test were applied to compare overall survival between the high-score and low-score groups by using the ‘survminer’ R package. To assess the predictive accuracy of the TGF-β risk model, we performed receiver operating characteristic curves (ROC) analysis and calculated the area under the curve (AUC) by using the ‘timeROC’ R package. Finally, the prediction role of TGF-β risk model was further validated in GSE121720 dataset.

#### Nomogram construction and validation

The Cox regression analyses were performed to determine the independent prognostic predictors. Then, we incorporated TGF-β risk score and clinical characters to construct a combined prognostic nomogram for GBM by using the ‘rms’ R package. The nomogram performance was quantified by ROC curves and calibration plots.

#### Gene mutation pattern analysis

Genomic variants, including single nucleotide variations (SNVs) in TCGA-GBM dataset were analyzed by using the ‘maftools’ R package. Wilcoxon test was applied for comparing the tumor mutation burden (TMB) and number of mutated genes, and Chi-square test was performed to demonstrate differences in mutational frequency between the two groups. The top ten genes with the highest mutational frequency were presented with waterfall plot.

#### Evaluation of TME immune characteristics

Based on the RNA expression data of TCGA-GBM dataset, single sample gene set enrichment analysis (ssGSEA) approach of the ‘GSVA’ R package was applied to evaluate the degree of immune cell infiltration in the TME, and the ESTIMATE algorithm was introduced to calculate the ESTIMATE score, stromal score, immune score, and tumor purity (21, 22). Then, we used ssGSEA procedure to quantify the enrichment scores of 17 immunotherapy related pathways (23). In addition, we also compared the difference in anticancer immune cycle in TME between two groups (24).

#### Prediction of the clinical response to immunotherapy/chemotherapy

The ‘pRRophetic’ R package was used to evaluate the clinical response to chemotherapeutics or targeted therapies, and the difference in the 50% inhibitory concentration (IC50) of the chemotherapy or targeted drugs were compared between the two groups (25). The patients’ response to immune checkpoint blockade (ICB) therapy was evaluated based on immunotherapy associated PRJNA482620 cohort (26).

#### Statistical analysis

All statistical analyses were performed with R software (Version: 4.1.2). Univariate and multivariate Cox regression analyses were used to identify independent risk factors for prognosis. We performed ROC analysis and calculated the AUC to assess the predictive accuracy of the TGF-β risk model. Continuous variables were compared between groups using the Wilcoxon test. Two-sided *P* less than 0.05 were considered statistically significant.

## Results

### Screening of TGF-β DEGs

**Figure 1:**
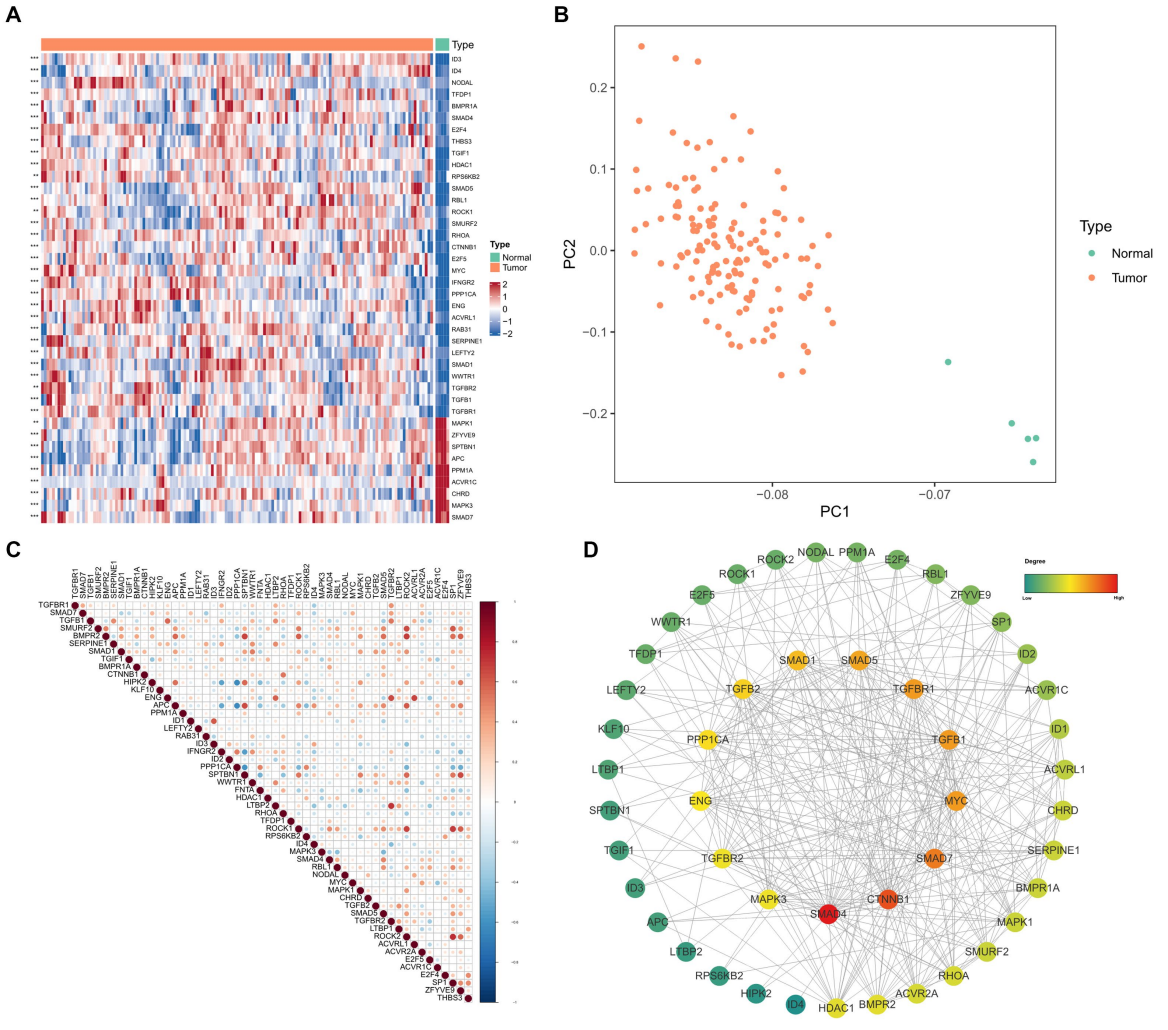
The differential expression and functional analysis of TGF-β genes in TCGA-GBM database. **(A)** Heatmap of the top 40 significantly differential TGF-β genes between tumor and normal samples. **(B)** PCA for tumor and normal samples. **(C)** Pearson correlation analysis of TGF-β DEGs. **(D)** PPI network analysis of TGF-β DEGs.

According to the thresholds mentioned above, 52 TGF-β DEGs were identified between GBM and normal samples. 39 TGF-β genes were overexpressed, and 13 genes were down-regulated in GBM samples. As shown in Figure 1A, ID3, ID4, and TGFβ1 genes were expressed significantly higher in GBM samples; while MAPK1, SMAD7, and APC genes were expressed significantly higher in normal samples. Principal component analysis (PCA) based on these 52 TGF-β DEGs showed excellent separation amongst tumor and normal samples (Figure 1B). Then we analyzed the correlation between TGF-β DEGs and GBM patients’ clinical features, and the results found that ACVR2A, WWTR1, ACVR1C, and TGFβ2 were differentially expressed in subgroups according to the age and gender, which indicating the sex-dimorphic and age-dependent expression of some TGF-β related genes in human GBM (Supplementary Figure S2). Based on the expression data, we also calculated the Pearson correlation of the TGF-β DEGs (Figure 1C). The PPI network revealed that SMAD4, CTNNB1, and SMAD7 may play critical roles in TGF-β signaling pathway in GBM (Figure 1D).

### Construction and validation of TGF-β risk model for GBM

**Figure 2:**
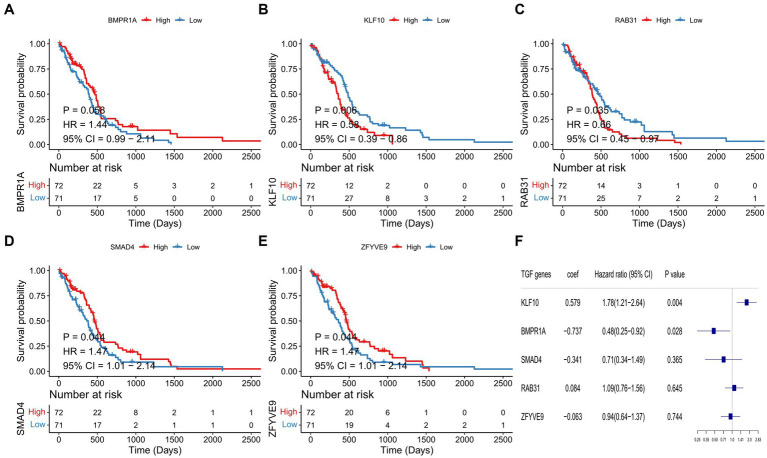
Identification of prognostic TGF-β genes and establishment of risk score model. **(A–E)** Kaplan–Meier survival curves for five prognostic genes in TCGA-GBM samples. **(F)** The multivariate Cox analysis of five prognostic genes.

First, five prognosis-related genes were identified from the 52 DEGs in TCGA-GBM dataset by univariate Cox regression analysis, including BMPR1A, KLF10, RAB31, SMAD4, and ZFYVE9. To assess the prognostic function of identified genes in GBM, survival curves were plotted based on median expression and compared by log-rank analysis (Figures 2A–E). Second, we performed multivariate Cox regression based on the selected five prognostic genes (Figure 2F). Finally, TGF-β risk model for GBM was developed according to the expression and coefficients of these five TGF-β genes.

**Figure 3:**
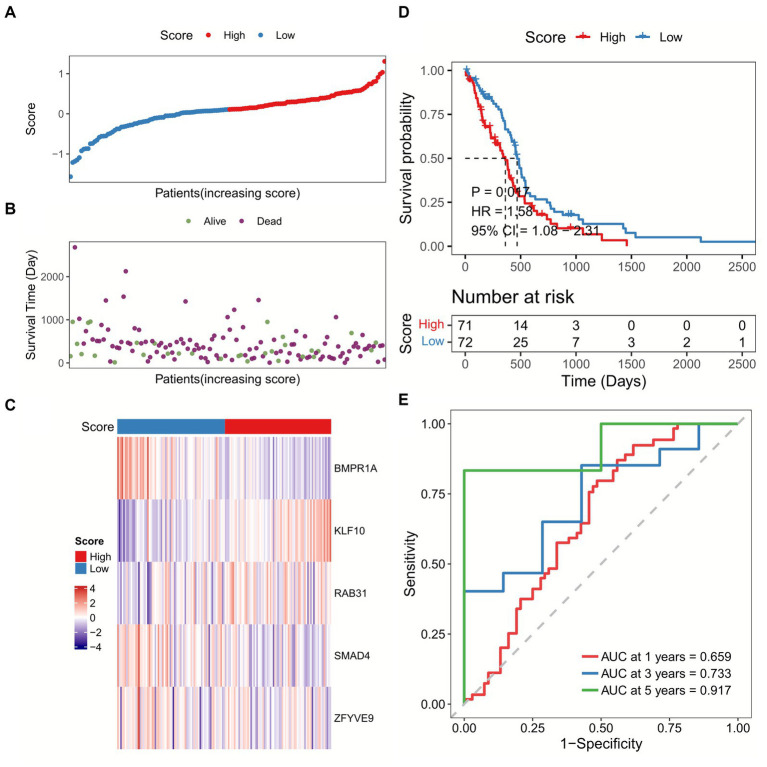
Development of a TGF-β risk score model in TCGA-GBM dataset. **(A)** The distribution of scores for each sample. **(B)** The different survival status of samples. **(C)** The expression of five prognostic genes for each sample. **(D)** Overall survival analysis between high and low-score groups. **(E)** ROC and AUC analysis of the TGF-β risk score.

**Figure 4:**
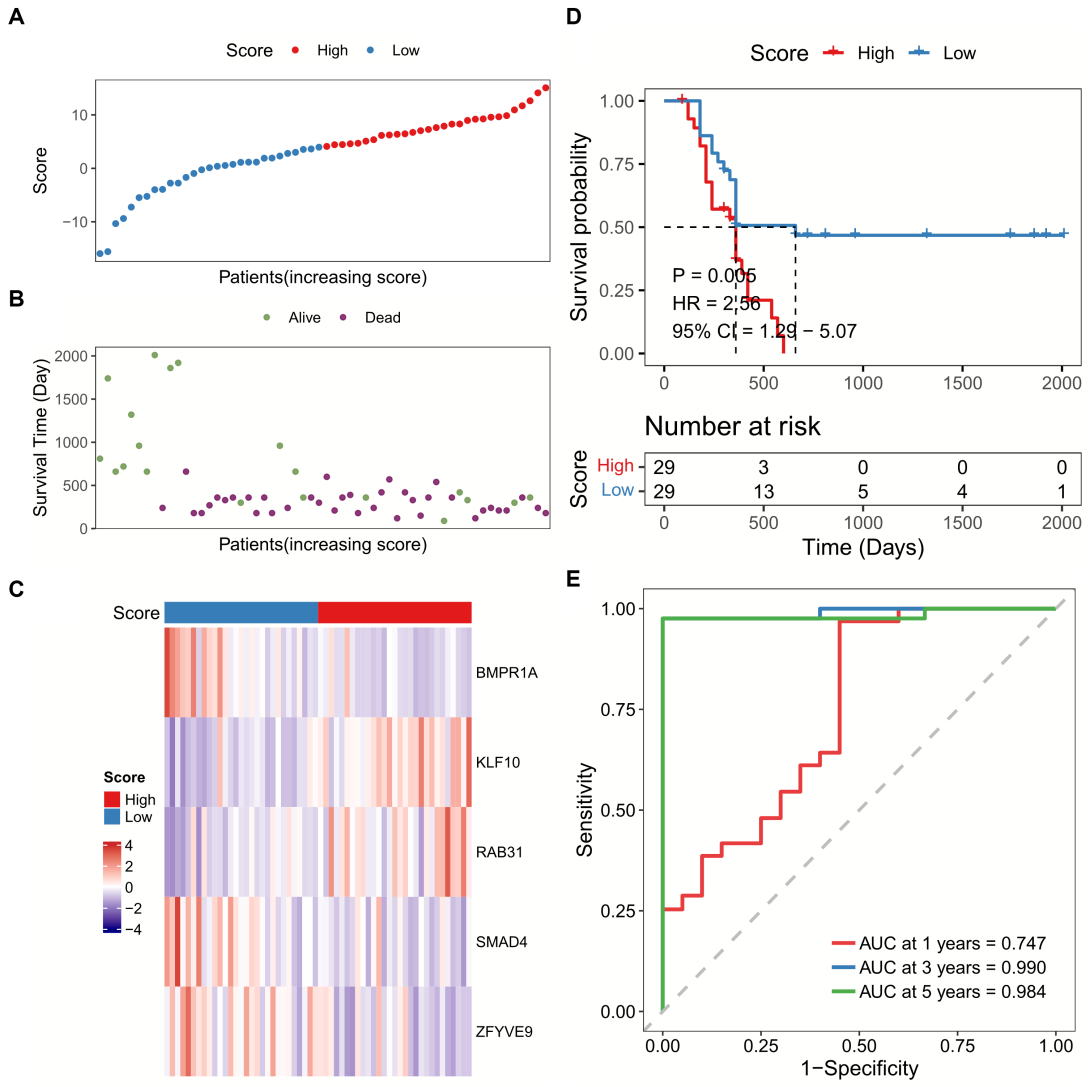
Validation of the TGF-β risk model in GSE121720 cohort. **(A)** The distribution of scores for each sample in GSE121720 cohort. **(B)** The different survival status of samples in GSE121720 cohort. **(C)** The expression of five prognostic genes for each sample. **(D)** Overall survival analysis between high and low-score groups. **(E)** ROC and AUC analysis of the TGF-β risk score.

We calculated the sample risk score in the TCGA-GBM dataset, and the patients were divided into high-score and low-score groups (Figures 3A–C). GBM patients with higher score had worse overall survival (OS) compared with patients with lower score (Figure 3D). Besides, the ROC analysis indicated that the AUC of TGF-β risk model in predicting 1-year, 3-year, and 5-year OS was 0.659, 0.733, and 0.917, respectively (Figure 3E). Furthermore, the TGF-β risk model was also introduced to calculate the risk score of validation cohort (Figures 4A–C), and the robustness of the TGF-β risk model was supported in GSE121720 cohort (Figure 4D). The ROC curves indicated that TGF-β risk score harbored a high level of AUC value for predicting 1/3/5-year OS with 0.747, 0.990, and 0.984, respectively (Figure 4E).

### Correlation between TGF-β risk score and clinical characteristics

**Figure 5:**
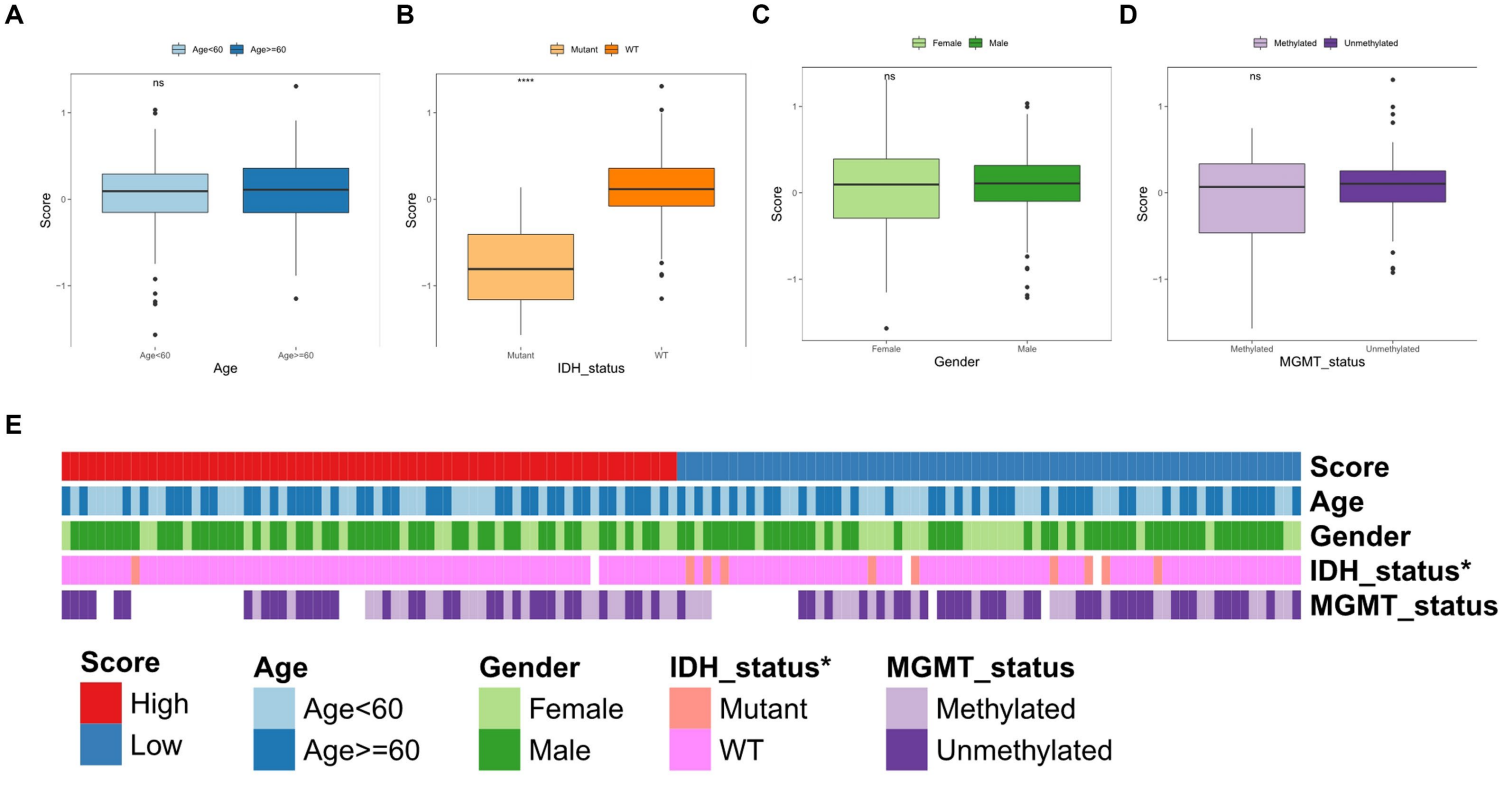
Correlation between TGF-β risk score and clinical features. **(A–D)** The difference of risk score between different clinical features including age, IDH status, gender, and MGMT status. Wilcoxon test was conducted. **(E)** The differences of clinical characteristics between two score groups.

We subsequently correlated the TGF-β risk model with the clinical features, such as age, gender, O-6-methylguanine-DNA methyltransferase (MGMT) status, and isocitrate dehydrogenase (IDH) status. There was no significant difference in the TGF-β risk score among subgroups according to age, gender, and MGMT status (Figures 5A,C,D). Patients with wild-type IDH had higher risk score than patients with mutant IDH (Figure 5B). In addition, the results also showed there was significant differences in the IDH mutation status between the two groups (Figure 5E).

### Development and validation of the nomogram

**Figure 6:**
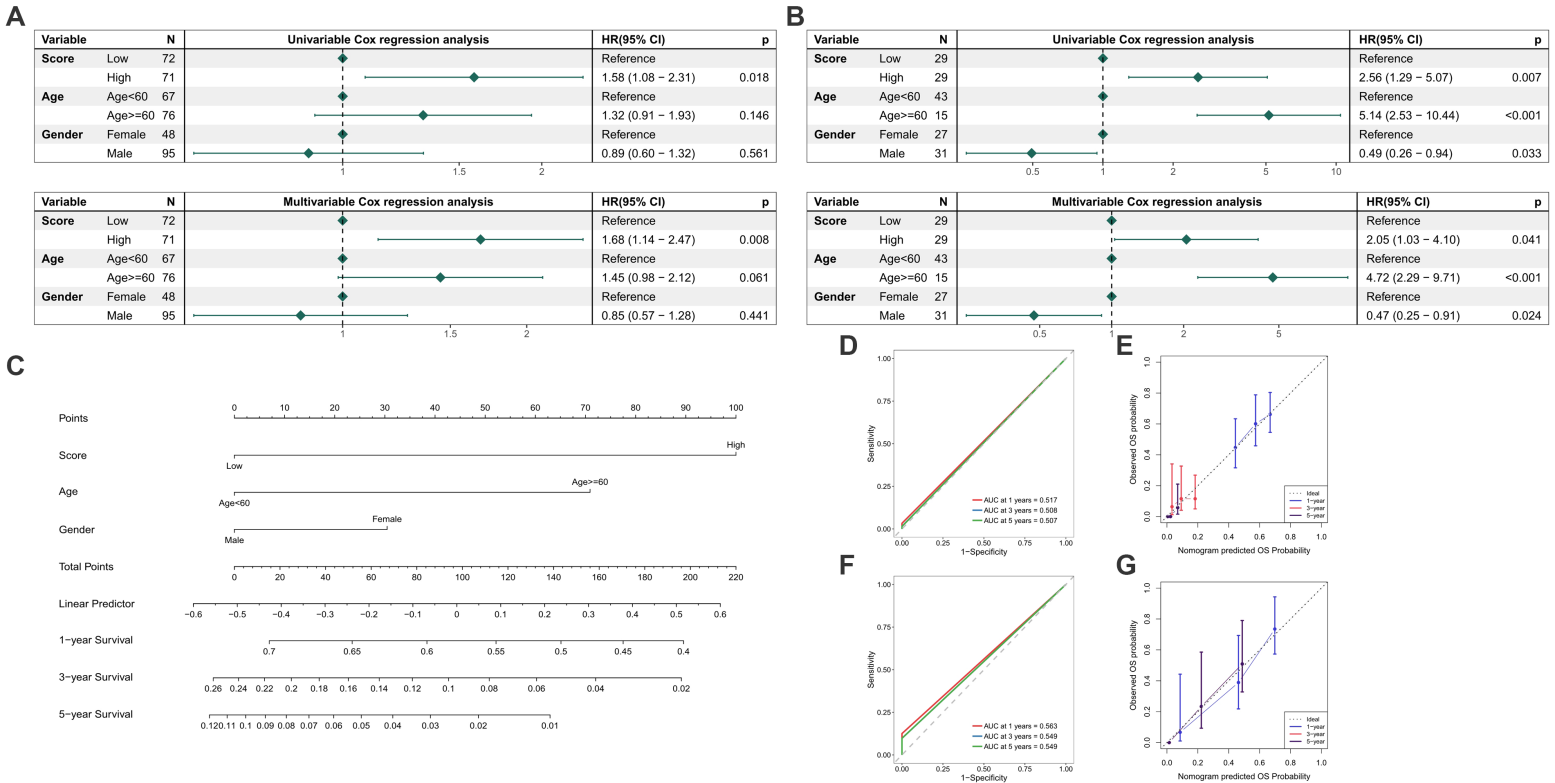
Development and validation of a comprehensive nomogram. **(A)** Results of Cox regression analyses in TCGA-GBM cohort. **(B)** Results of Cox regression analyses in GSE121720 cohort. **(C)** Nomogram construction based on age, gender, and TGF-β risk score. **(D,E)** The ROC curves and calibration plot of the nomogram in TCGA-GBM dataset. **(F,G)** The ROC curves and calibration plot of the nomogram in GSE121720 dataset.

The Cox regression analyses found that TGF-β risk score was a prognostic factor in TCGA-GBM cohort (Figure 6A). The GSE121720 cohort further confirmed TGF-β risk score as an independent prognostic factor for GBM (Figure 6B). Then, we incorporated the TGF-β risk score with gender and age to construct a combined nomogram (Figure 6C). The ROC analysis indicated that the AUC of nomogram in predicting 1-year, 3-year, and 5-year OS was 0.517, 0.508, and 0.507, separately (Figure 6D). The calibration plot verified that the predicted survival using nomogram was strongly consistent with actual OS (Figure 6E). The ability of the nomogram in predicting prognosis was further assessed in the GSE121720 cohort (Figures 6F,G).

### TGF-β risk score and genetic variation

**Figure 7:**
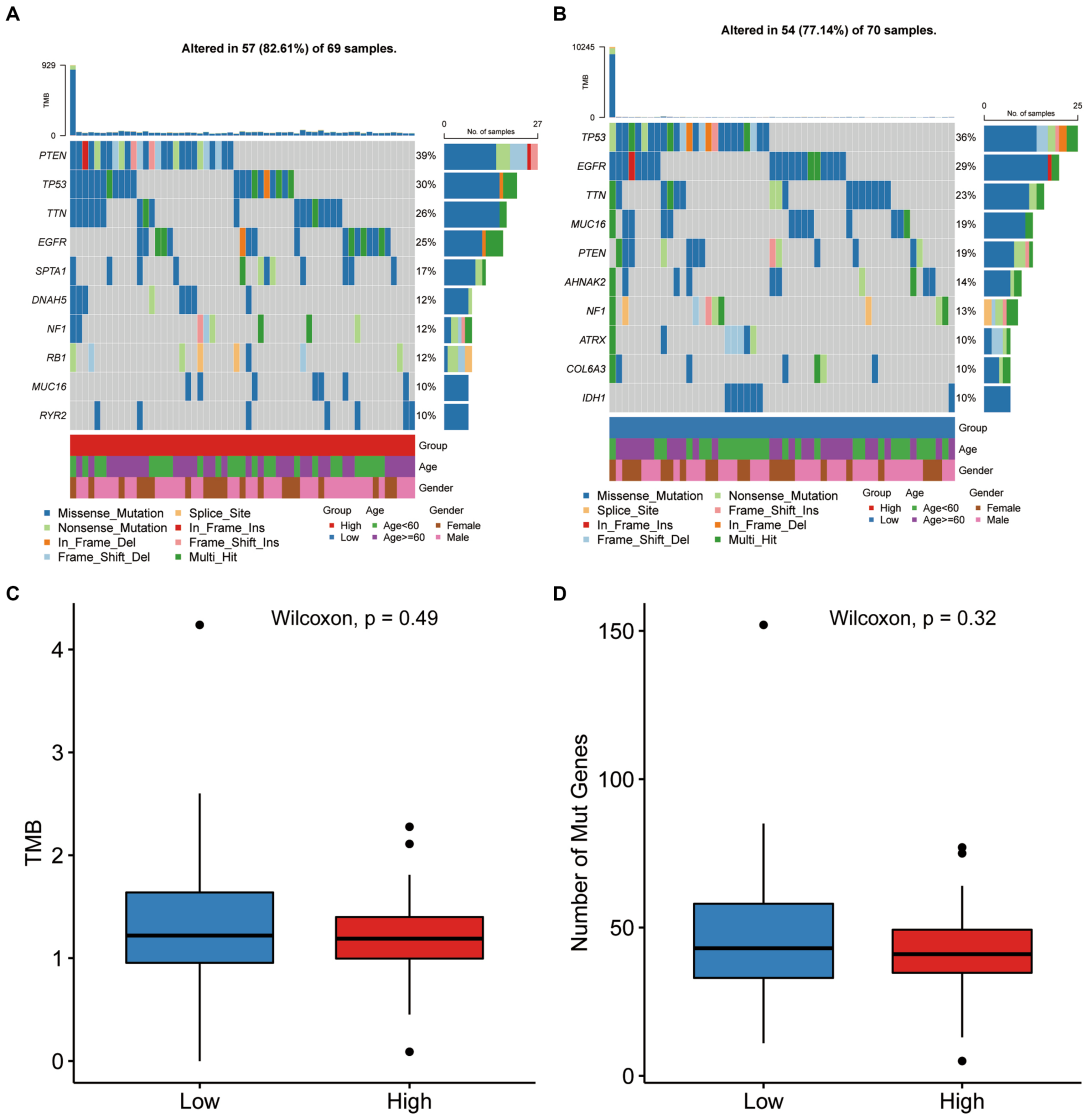
Gene mutation differences between the two score groups. **(A,B)** The waterfall plot showing the mutation distribution of the top ten mutated genes in high-score and low-score group. **(C,D)** Comparison of TMB and number of mutated genes between high-score and low-score group.

In these two risk groups, the top ten genes with the highest mutation frequencies were depicted in Supplementary Figure S3. Between the two groups, statistical analysis of Chi-square showed that mutation frequencies of SNVs in eight genes were significantly different, including PTEN, SPTA1, DNAH5, IDH1, MROH2B, CDH9, AHNAK2, and TENM4 (Figures 7A,B). Wilcoxon test was also applied for comparing the TMB and number of mutated genes between high-score and low-score groups, and no significant difference in TMB and the number of mutations were observed between the two groups (Figures 7C,D).

### TGF-β risk model predicted the TME immune features

**Figure 8:**
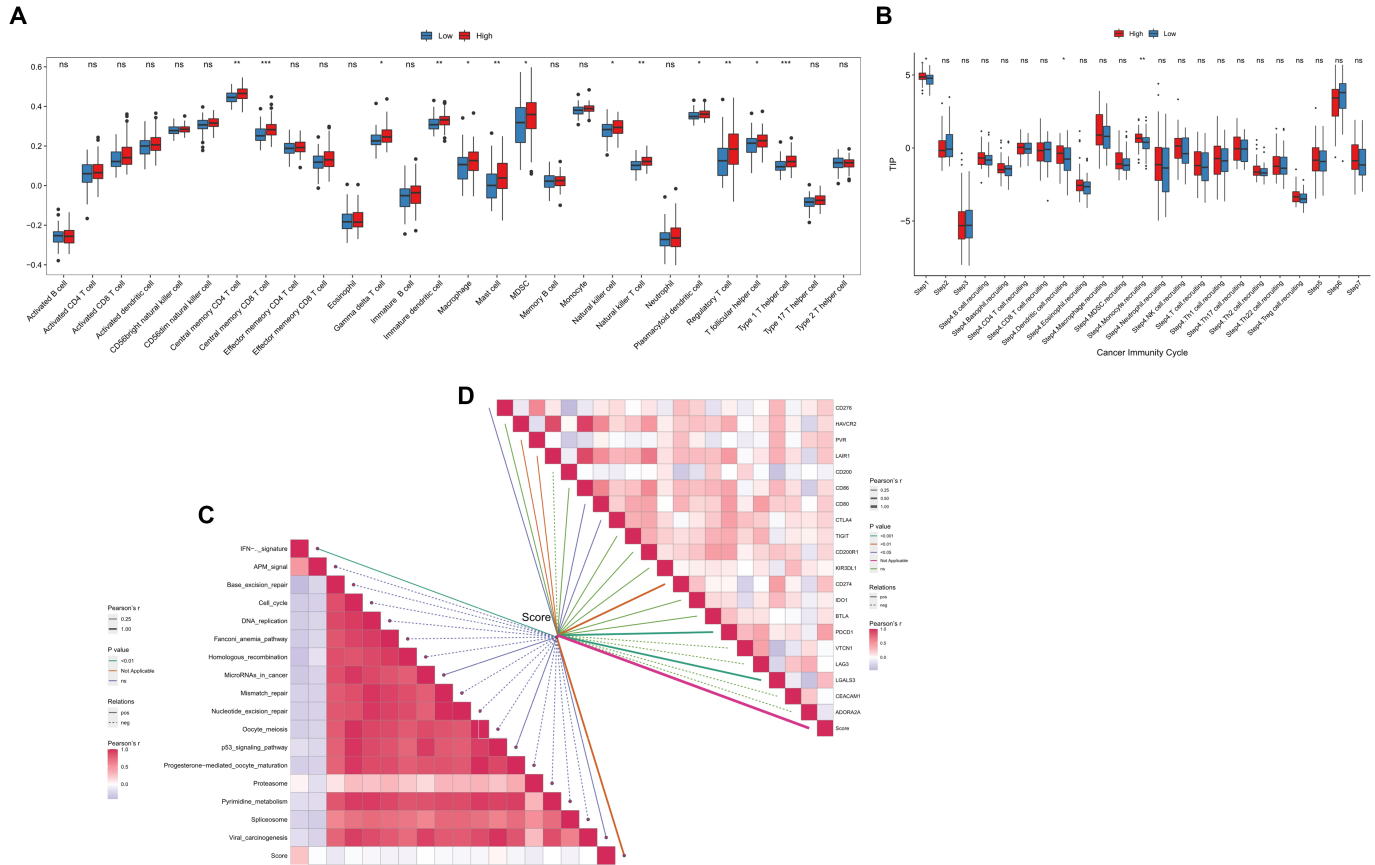
Relationships between the TGF-β risk score and TME immune characteristics. **(A)** The TGF-β risk score and the infiltration levels of 28 immune cells. **(B)** The TGF-β risk score and the enrichment of anticancer immunity steps. **(C)** The TGF-β risk score and the enrichment of immunotherapy related pathways. **(D)** The TGF-β risk score and the expression of immune checkpoints.

The TME status could affect the survival and immunotherapy sensitivity of cancer patients (27, 28). First, the ssGSEA analysis showed that the infiltration levels of several immune cells, including central memory CD8 T cell, macrophage, immature dendritic cell, mast cell, and natural killer cells, were positively correlated with the TGF-β risk score (Figure 8A). Second, the enrichment scores of cancer cell antigens release, dendritic cells and monocytes recruiting were significantly higher in high-score group (Figure 8B). In addition, the TGF-β risk score had a tight association with enrichment score of immunotherapy response related pathway, including IFN-γ signature (Figure 8C). Furthermore, eight immune checkpoints were significantly correlated with the TGF-β risk score, including CD274 (PD-L1), PDCD1, CTLA4, CD276, PVR, CD80, LAIR1, LGALS3 (Figure 8D). To better understand the difference of TME status between the high-score and low-score groups, ESTIMATE analysis was carried out. The ESTIMATE, stromal and immune scores of the high-score group were higher than those of the low-score group, while tumor purity was significantly lower in the high-score group than low-score group (Supplementary Figure S4). Collectively, the TGF-β risk score predicted TME immune status for GBM.

Additionally, we analyzed the tight associations between the TGF-β risk score and the expression of TGF-β family members, TGF-β family members receptors, and immune checkpoints. The results displayed a remarkable difference in the expression of GDF15, BMP1, TGFβ1, INHBB, LEFTY1, and BMPR1A between the high-and low-score groups (Supplementary Figures S5, S6). Besides, the TGF-β risk score positively correlated with several immune checkpoints, such as CD28, CD96, IL2RA, PDCD1, TIGIT, PVR (Supplementary Figure S7).

### TGF-β risk score predicted the response to immunotherapy and chemotherapy

**Figure 9:**
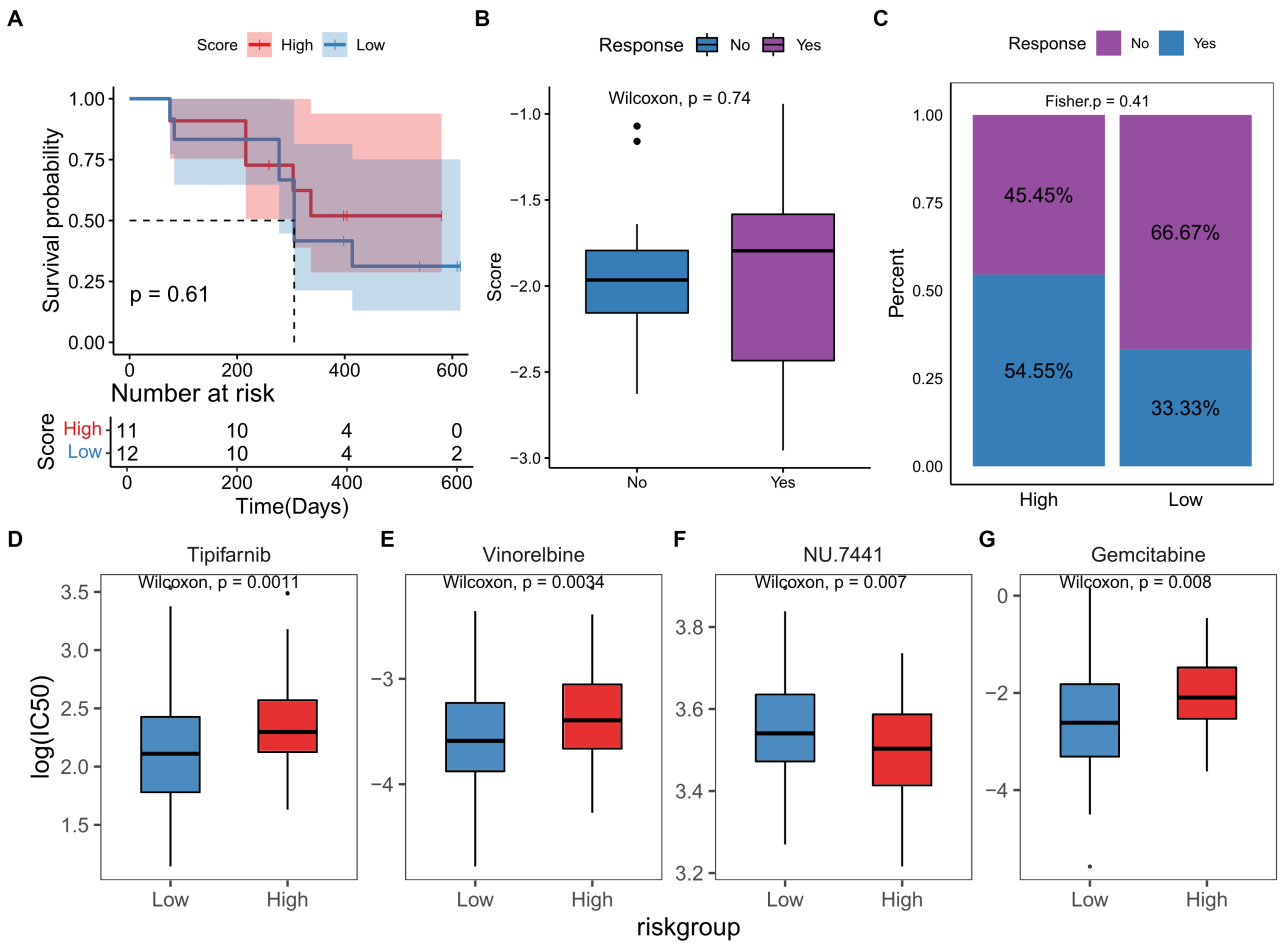
TGF-β risk model predicted the clinical response of immunotherapy and chemotherapy. **(A)** Survival difference between high-and low-score groups in immunotherapy cohort. **(B)** Comparison of risk scores in patients with and without response to immunotherapy. **(C)** Proportion of patients with and without response to immunotherapy in two score groups. **(D–G)** The differences in responses to chemotherapy drugs between two score groups.

In the PRJNA482620 cohort, we did not find any significant associations between the TGF-β risk score and GBM prognosis (Figure 9A). There was no significant difference in the risk scores in patients with and without response to immunotherapy (Figure 9B). The group of GBM patients with high score showed a higher response to ICB treatment, though this difference was not significant (Figure 9C). All patients in PRJNA482620 cohort received the standard therapy of temozolomide and radiation before immunotherapy (26), which may partially diminish the relationship between risk score and immunotherapy response.

Based on the RNA sequencing data of TCGA-GBM dataset, the IC50 of chemotherapy or targeted drugs was estimated, and we observed that high-score GBM samples were more sensitive to NU.7441, and low-score samples were more sensitive to tipifarnib, vinorelbine, and gemcitabine (Figures 9D–G).

## Discussion

The TGF-β signaling pathway exhibits both tumor-suppressing and tumor-promoting roles (29, 30). In addition, TGF-β is a immune suppressive mediator, inhibiting antigen presentation cells and effector T cells (31). In consideration of the critical roles of TGF-β in regulating tumor biology, increasing efforts have been made to the therapeutic potentials of TGF-β. Previous studies have shown that TGF-β has been closely implicated in the GBM progression. For instance, TGF-β1 promoted enhanced microtubes formation and communication via calcium signaling in GBM cell lines, and targeting TGF-β pathway could serve as a new method against microtubes mediated invasion/resistance (32). TGF-β induced a shift in metabolism from oxidative phosphorylation to aerobic glycolysis, suppressing antitumor immunity and facilitating GBM progression (33). However, little work has explored the function of TGF-β signature in predicting prognosis and regulating immune status in GBM. In this study, we firstly screened differential TGF-β genes in the TCGA-GBM cohort. Then, five optimal genes were selected to develop a TGF-β risk model according to the expression and coefficients of these five genes, including BMPR1A, KLF10, RAB31, SMAD4, and ZFYVE9.

BMPR1A was progressively expressed in malignant glioma and promoted tumorigenesis in a murine model of glioma (34). RAB31 inhibited apoptosis, and promoted proliferation and migration in U87 cells (35). The reduced expression of SMAD4 was associated with poor outcome of glioma patients and SMAD4 exerted an inhibitory role in glioma development (36). The increased expression of Stanniocalcin-1 may promote the progression of glioblastoma via the TGF-β/SMAD4 pathway (37). Several studies have defined KLF10 as a tumor suppressor, inducing apoptosis and inhibiting proliferation in various cancer cells (38). ZFYVE9 has been reported to be dispensable for functional TGF-β mediated signaling (39). To date, the expression and function of KLF10 and ZFYVE9 in GBM have not been studied. Then, a novel TGF-β risk model with independent prognostic prediction value was developed based on these five genes.

TGF-β is an essential immune-suppressive cytokine in the TME, and TGF-β production by tumor cells promotes tumor growth and immune escape (11, 12). We verified that the TGF-β risk model could predict the TME immune status. First, the TGF-β risk score had significant positive correlations with the infiltration levels of tumor-infiltrating immune cells, and the scores of anticancer immunity steps. As a result, the anticancer immune response was more activated in the TME, and the anticancer activity was higher in GBM patients with high-score. Second, we found the IFN-γ signature was significantly enriched in high-score patients. Besides, the TGF-β risk score positively correlated with several immune checkpoints, including PDCD1, PD-L1, and CTLA4. The results demonstrated that the TME with higher score indicated higher sensitivity to ICB and better effect of immunotherapy. However, the results also revealed that the risk score correlated with the infiltration levels of myeloid-derived suppressor cells and regulatory T cells, which were considered to inhibit anticancer immunity (40).

An increasing number of risk models related to pyroptosis, autophagy, ferroptosis, and glucose metabolism have been constructed with the potential value to predict prognosis and guide individualized treatment for GBM (41–43). Several studies investigating the prognostic prediction in tumors of TGF-β signature have been published, such as hepatocellular carcinoma, lung adenocarcinoma, and bladder cancer (44–46). In this study, the optimal candidate genes were firstly screened from TGF-β related genes with several programs, including univariate and multivariate Cox analyses. Second, we developed a five-gene signature model and correlated it with clinical features, genetic variation, immune phenotypes, and therapeutic response. Then, we validated our risk model in external cohort. Nevertheless, there were some deficiencies in current study. First, our present results were obtained merely based on public databases, and therefore *in vitro* and *in vivo* experiments are needed to verify these results. Second, only one external cohort was applied to validate our results, and the results remain to be further proven with a large-scale, prospective study. Third, the potential application in clinical practice of the TGF-β signature requires more effort.

In conclusion, we successfully constructed a TGF-β risk model with an independent prognostic prediction value for GBM. Patients with high-score had worse prognosis than patients with low-score. The TGF-β signature was closely correlated with the clinical features, genetic variation, and TME immune status of GBM. It also predicted the sensitivity to therapeutic approach for GBM.

## Data availability statement

The original contributions presented in the study are included in the article/Supplementary material, further inquiries can be directed to the corresponding author.

## Author contributions

HL and ZW: conception and design. ZW, KS, and YZ: provision of study materials. ZW, KS, and JL: collection and assembly of data. HL, ZW, and JL: data analysis and interpretation. HL: manuscript writing. All authors contributed to the article and approved the submitted version.

## Funding

This work was supported by the grants from the Medical Key Cultivation Discipline Program of Luoyang City (STE-2022-5).

## Conflict of interest

The authors declare that the research was conducted in the absence of any commercial or financial relationships that could be construed as a potential conflict of interest.

## Publisher’s note

All claims expressed in this article are solely those of the authors and do not necessarily represent those of their affiliated organizations, or those of the publisher, the editors and the reviewers. Any product that may be evaluated in this article, or claim that may be made by its manufacturer, is not guaranteed or endorsed by the publisher.

## Supplementary material

SUPPLEMENTARY FIGURE S1 The workflow of TGF-β Based Risk Model for GBM.

SUPPLEMENTARY FIGURE S2 The correlation between the differentially expressed TGF-β genes and GBM patients’ clinical features in TCGA database.

SUPPLEMENTARY FIGURE S3 Gene mutation features in TCGA-GBM dataset.

SUPPLEMENTARY FIGURE S4 The ESTIMATE, immune and stromal scores, and tumor purity of two risk groups.

SUPPLEMENTARY FIGURE S5 Differentially expressed TGF-β family members between the high- and low-score groups.

SUPPLEMENTARY FIGURE S6 Differentially expressed TGF-β family members receptors between the high- and low-score groups.

SUPPLEMENTARY FIGURE S7 Differentially expressed immune checkpoints genes between the high- and low-score groups.

SUPPLEMENTARY TABLE S1 The clinical characteristics of patients in included dataset.

SUPPLEMENTARY TABLE S2 A list of 121 TGF-β related genes.
