# Supplementary figures and images for: TGF-β based risk model to predict the prognosis and immune features in glioblastoma
Source: Front Neurol. 2023 Jun 29;14:1188383. doi: 10.3389/fneur.2023.1188383 (PMC10343447; doi:10.3389/fneur.2023.1188383)

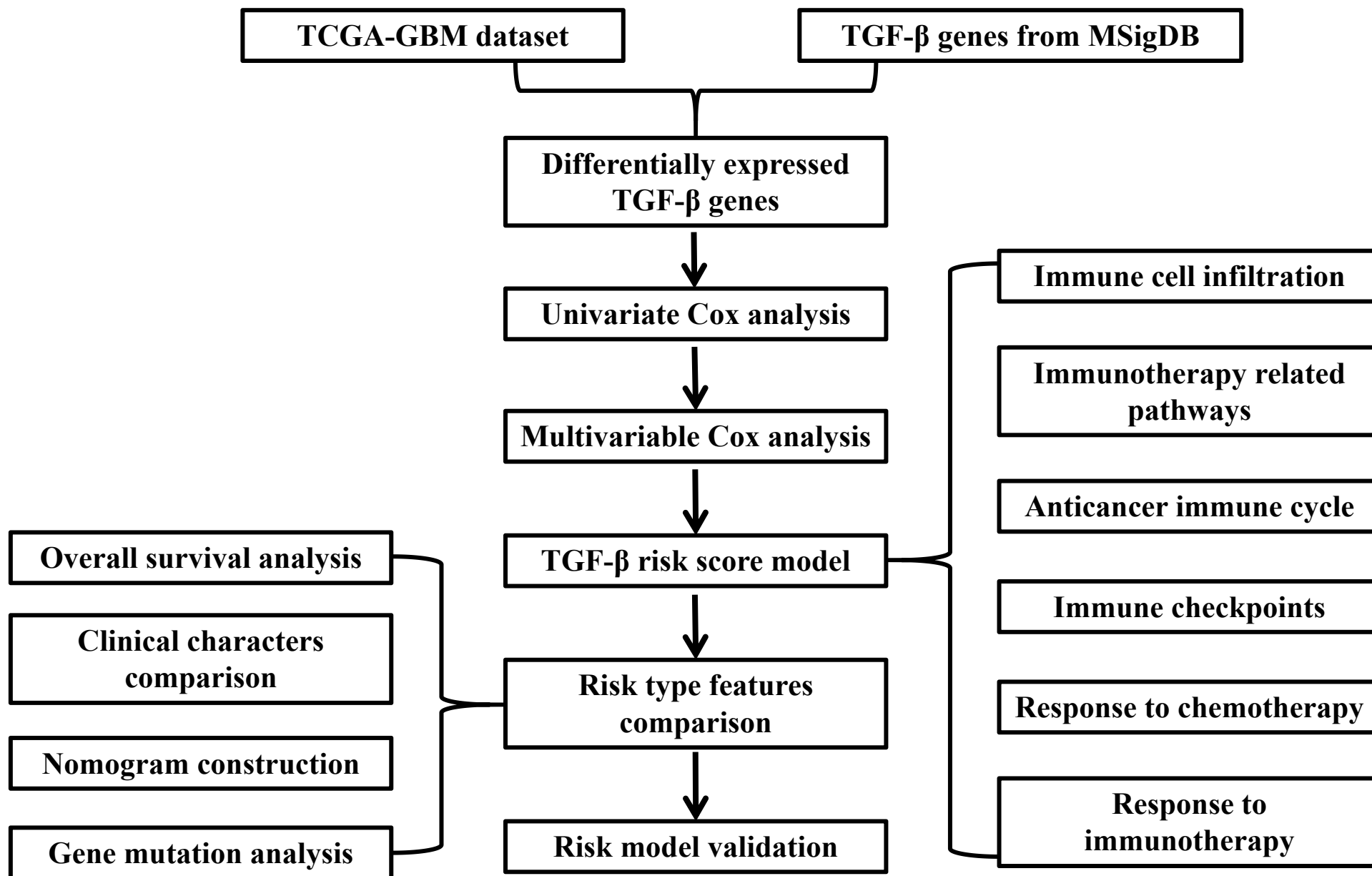

Supplement: SUPPLEMENTARY FIGURE S1 — The workflow of TGF-β Based Risk Model for GBM. [file Data_Sheet_1.PDF]

**A**

ACVR2A

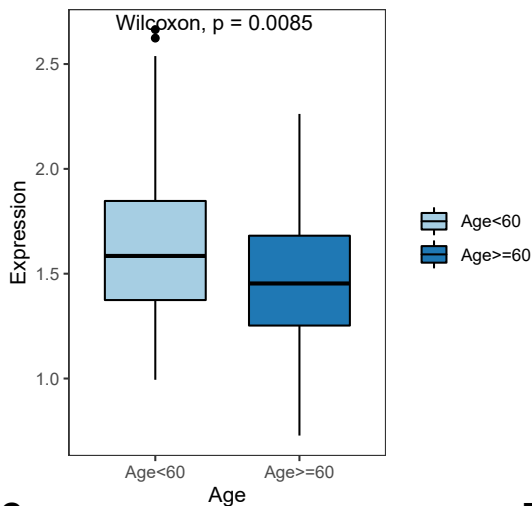**B**

WWTR1

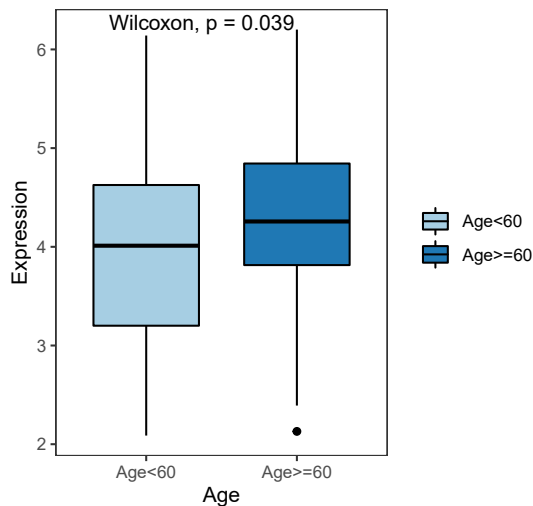**C**

ACVR1C

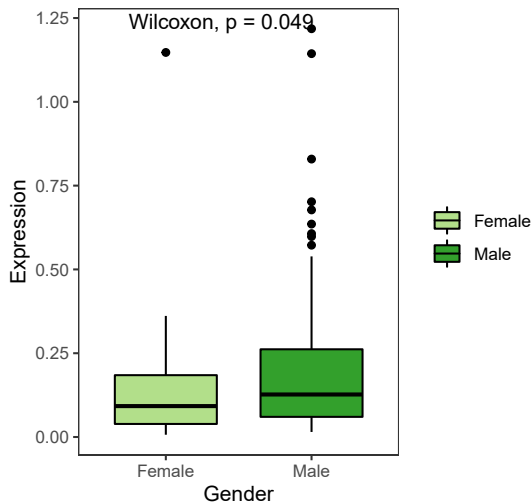**D**

TGFB2

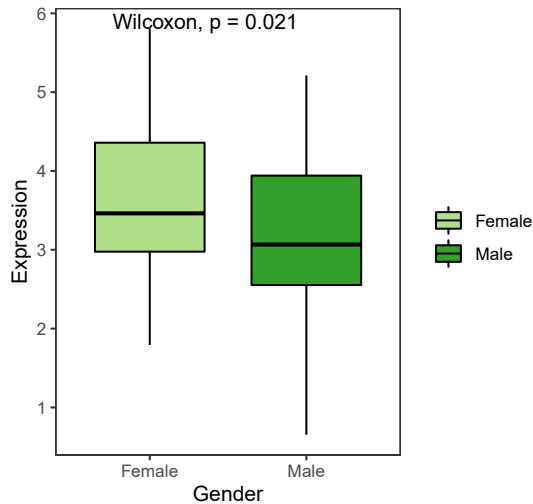

Supplement: SUPPLEMENTARY FIGURE S2 — The correlation between the differentially expressed TGF-β genes and GBM patients’ clinical features in TCGA database. [file Data_Sheet_2.PDF]

Altered in 108 (27.48%) of 393 samples.

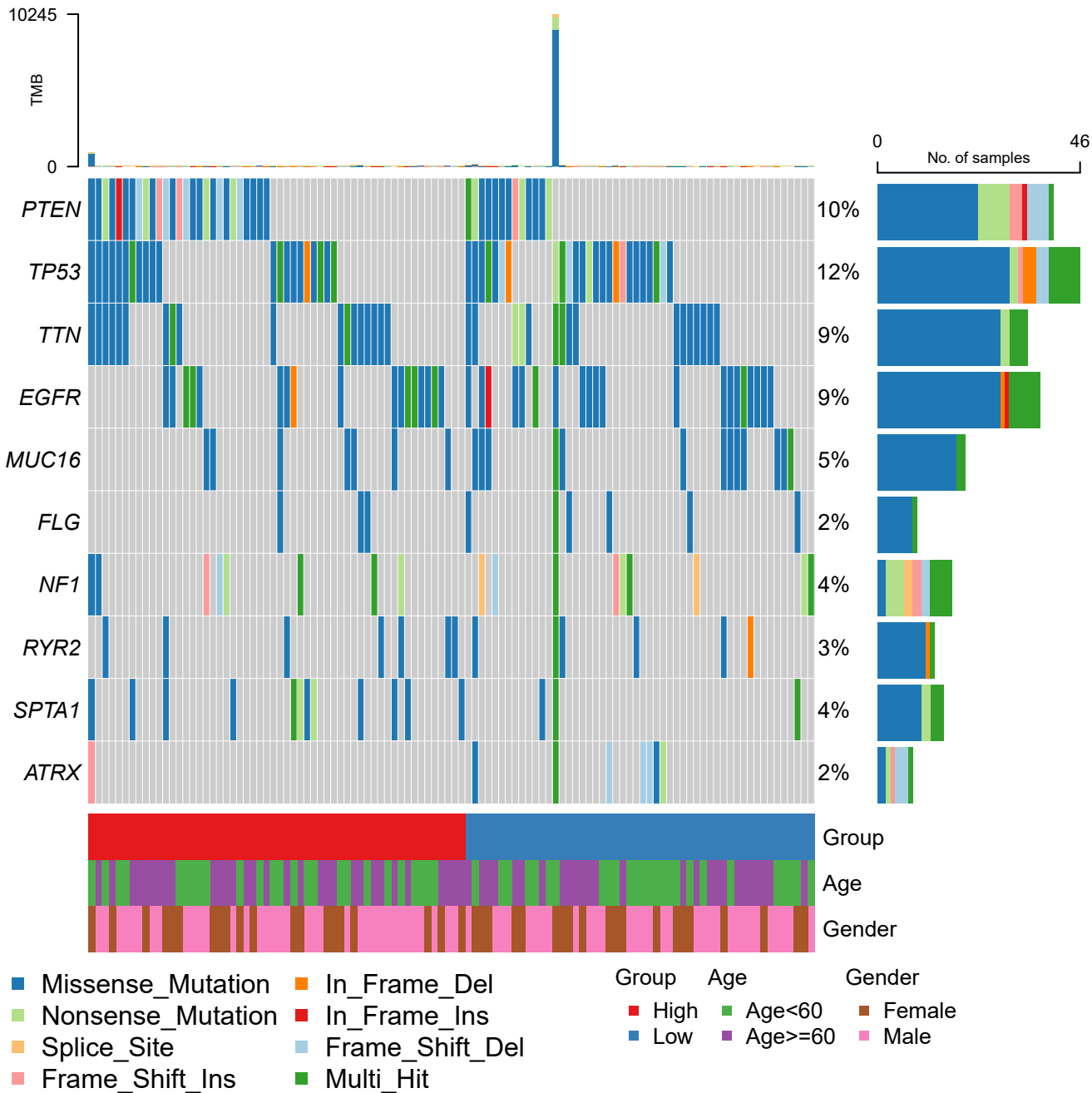

Supplement: SUPPLEMENTARY FIGURE S3 — Gene mutation features in TCGA-GBM dataset. [file Data_Sheet_3.PDF]

ESTIMATEScore

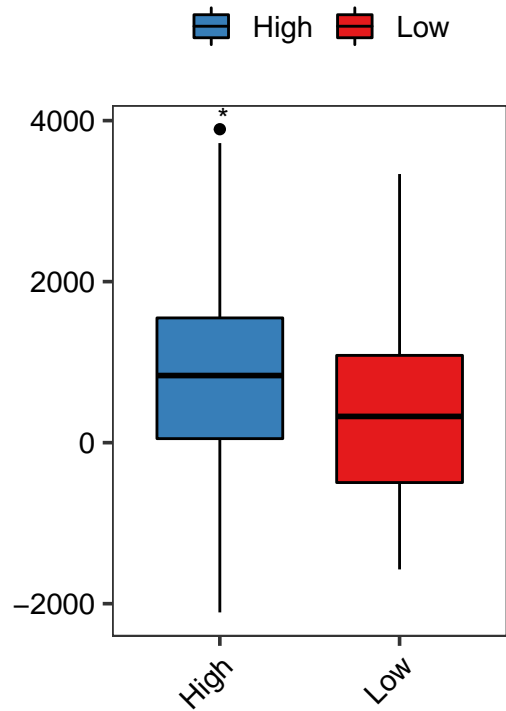

ImmuneScore

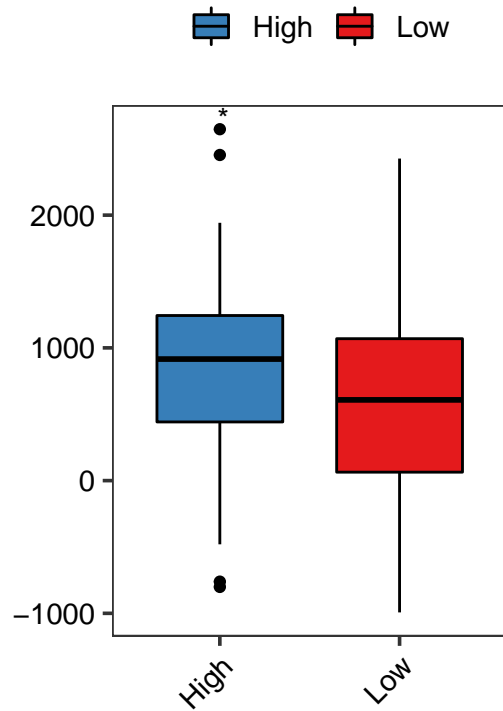

StromalScore

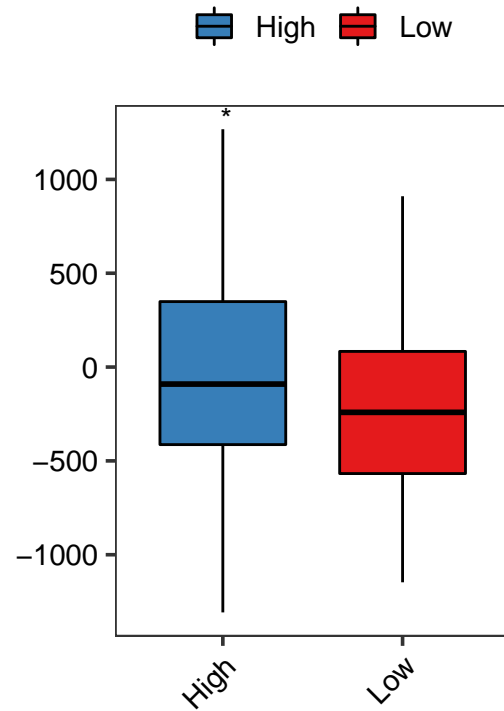

TumorPurity

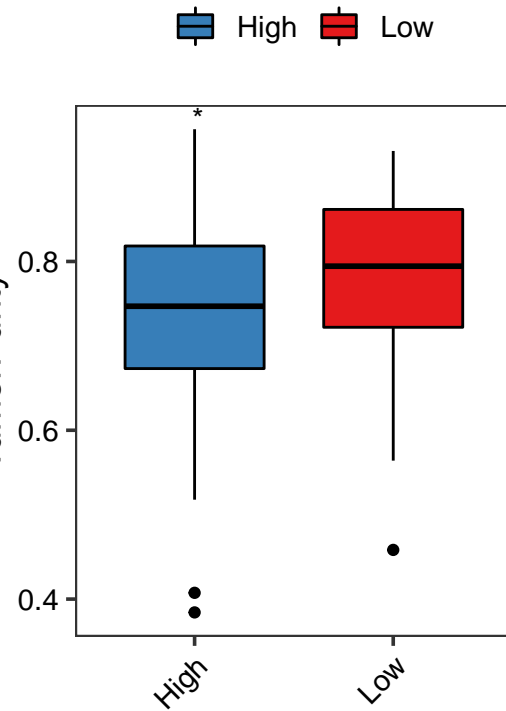

Supplement: SUPPLEMENTARY FIGURE S4 — The ESTIMATE, immune and stromal scores, and tumor purity of two risk groups. [file Data_Sheet_4.PDF]

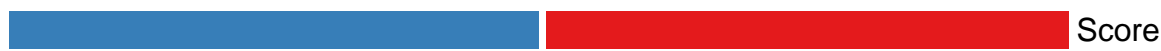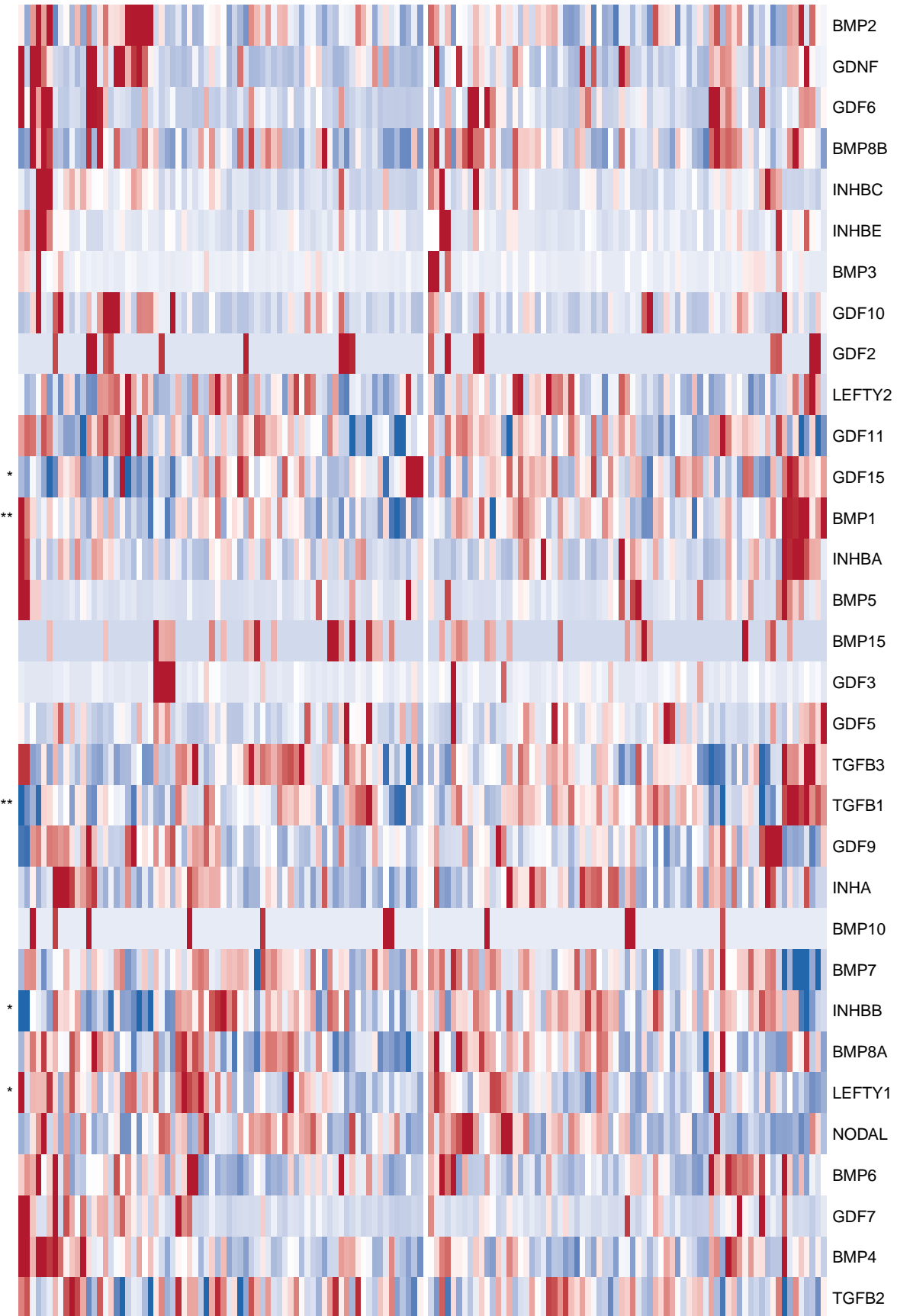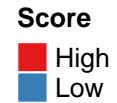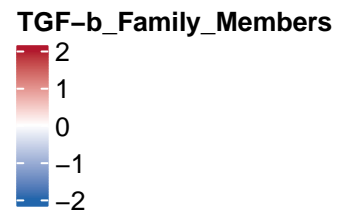

Supplement: SUPPLEMENTARY FIGURE S5 — Differentially expressed TGF-β family members between the high- and low-score groups. [file Data_Sheet_5.PDF]

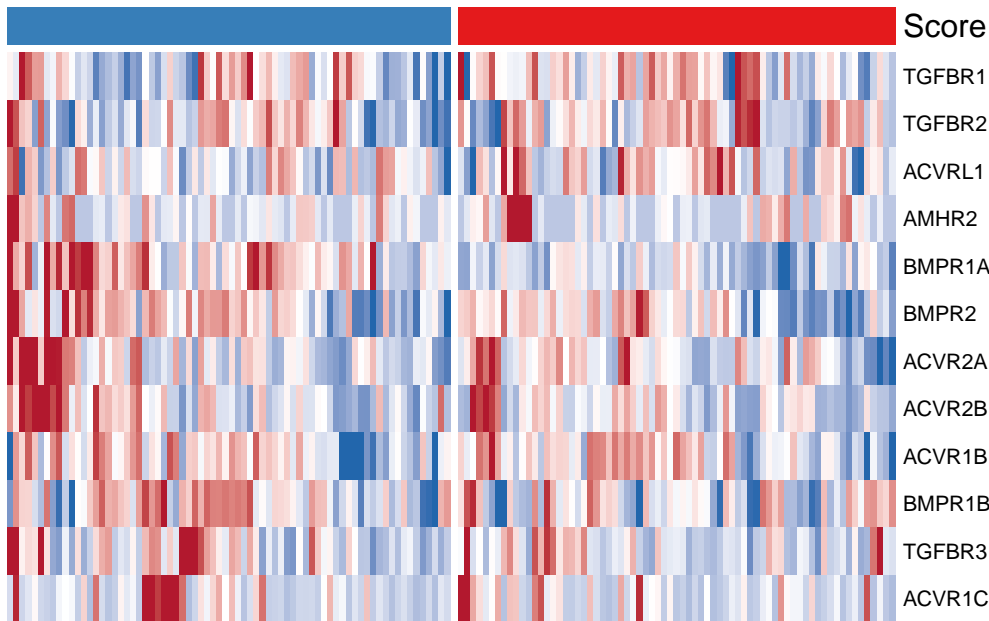

**Score**

High

Low

**TGF- $\beta$  Family Members Receptors**

2

1

0

-1

-2

Supplement: SUPPLEMENTARY FIGURE S6 — Differentially expressed TGF-β family members receptors between the high- and low-score groups. [file Data_Sheet_6.PDF]

Low

High

Score

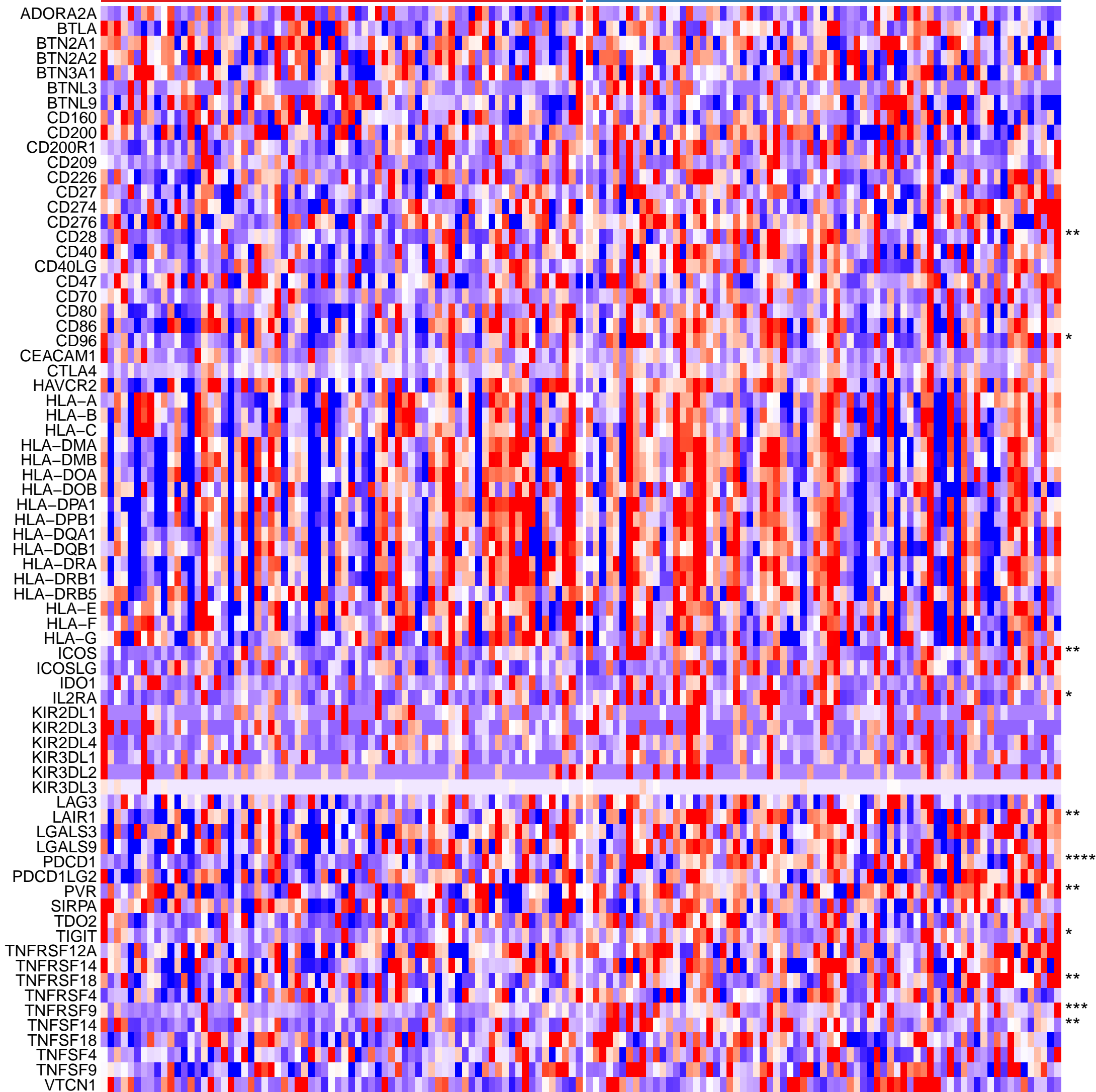

Score

Low  
High

Pvalue

ns \* &lt;0.05 \*\* &lt;0.01 \*\*\* &lt;0.001 \*\*\*\* &lt;0.0001

>=1  
0  
<=-1

Supplement: SUPPLEMENTARY FIGURE S7 — Differentially expressed immune checkpoints genes between the high- and low-score groups. [file Data_Sheet_7.PDF]
