# Supplementary material for: TGF-β based risk model to predict the prognosis and immune features in glioblastoma
Source: Front Neurol. 2023 Jun 29;14:1188383. doi: 10.3389/fneur.2023.1188383 (PMC10343447; doi:10.3389/fneur.2023.1188383)
Supplement: SUPPLEMENTARY TABLE S1 — The clinical characteristics of patients in included dataset. [file Table_1.pdf]

**Clinical feature**	**Value**	**Sample_num**	**Frequency (%)**
Age	Age<60	67	46.853
	Age>=60	76	53.147
Gender	Female	48	33.566
	Male	95	66.434
IDH_status	Mutant	10	6.993
	WT	130	90.909
	NA	3	2.098
MGMT_status	Methylated	46	32.168
	Unmethylated	67	46.853
	NA	30	20.979
