# Supplementary material for: TGF-β based risk model to predict the prognosis and immune features in glioblastoma
Source: Front Neurol. 2023 Jun 29;14:1188383. doi: 10.3389/fneur.2023.1188383 (PMC10343447; doi:10.3389/fneur.2023.1188383)
Supplement: SUPPLEMENTARY TABLE S2 — A list of 121 TGF-β related genes. [file Table_2.pdf]

TGFBR1
SMAD7
TGFB1
SMURF2
SMURF1
BMPR2
SKIL
SKI
ACVR1
PMEPA1
NCOR2
SERPINE1
JUNB
SMAD1
SMAD6
PPP1R15A
TGIF1
FURIN
SMAD3
FKBP1A
MAP3K7
BMPR1A
CTNNB1
HIPK2
KLF10
BMP2
ENG
APC
PPM1A
XIAP
CDH1
ID1
LEFTY2
CDKN1C
TRIM33
RAB31
TJP1
SLC20A1
CDK9

ID3
NOG
ARID4B
IFNGR2
ID2
PPP1CA
SPTBN1
WWTR1
BCAR3
THBS1
FNTA
HDAC1
UBE2D3
LTBP2
RHOA
TFDP1
TNF
GDF7
INHBB
INHBC
COMP
INHBA
THBS4
CREBBP
ROCK1
RPS6KB1
RPS6KB2
CUL1
SKP1P2
ID4
MAPK3
RBL2
SMAD4
RBL1
NODAL
MYC
SMAD2
MAPK1
EP300

BMP8A
GDF5
SKP1
CHRD
TGFB2
IFNG
CDKN2B
PPP2CB
PPP2CA
PPP2R1A
SMAD5
RBX1
FST
PITX2
PPP2R1B
TGFBR2
AMHR2
LTBP1
LEFTY1
AMH
SMAD9
ROCK2
TGFB3
GDF6
BMPR1B
ACVRL1
ACVR2B
ACVR2A
BMP4
E2F5
ACVR1C
E2F4
SP1
BMP7
BMP8B
ZFYVE9
BMP5
BMP6
ZFYVE16

THBS3
INHBE
THBS2
DCN
